# The β-hCG-TNF-α-T cell axis in threatened abortion: unraveling the pathogenic cascade from aberrant glycosylation to inflammatory demise

**DOI:** 10.3389/fimmu.2026.1749672

**Published:** 2026-08-28

**Authors:** Shimeng Wang, Qing Guo, Dingren Niu, Yao Chen, Yunlu Ping, Jiajie Ren, Ding Qi, Xiaoling Feng

**Affiliations:** 1 The 1st Affiliated Hospital of Heilongjiang University of Traditional Chinese Medicine, Harbin, Heilongjiang, China; 2 Heilongjiang Academy of Traditional Chinese Medicine, Harbin, Heilongjiang, China; 3 Heilongjiang University of Chinese Medicine, Harbin, Heilongjiang, China; 4 The 2nd Affiliated Hospital of Heilongjiang University of Traditional Chinese Medicine, Harbin, Heilongjiang, China

**Keywords:** danger signal hypothesis, immunometabolism, pyroptosis, threatened abortion, TNF-α, β-hCG glycosylation

## Abstract

Threatened abortion (TA), vaginal bleeding before 20 weeks in a viable pregnancy, affects 20-25% of early pregnancies. Its unpredictable nature is a major clinical challenge. Current diagnostics (β-hCG and ultrasound) are retrospective and fail to reveal the dynamic pathology. This review presents the “Danger Signal” Hypothesis, focusing on the β-hCG-TNF-α-T cell axis, to explain TA pathogenesis. The process involves three stages: 1) Initiation: Stressed trophoblasts secrete aberrantly glycosylated β-hCG, which acts as a DAMP. 2) Amplification: This DAMP triggers a TNF-α-dominated cytokine storm, reprogramming T cell metabolism to glycolysis and sustaining inflammation. 3) Execution: The inflammatory microenvironment induces trophoblast pyroptosis, creating a vicious cycle of damage. We synthesize evidence from glycobiology, immunometabolism, and cell death research to substantiate this model, explore its implications for the development of stage-specific biomarkers (such as β-hCG glycoforms and GSDMD-N fragments), and discuss precision therapeutic approaches (including the use of metformin for metabolic intervention and anti-TNF-α agents for managing late-phase inflammation). This mechanistic framework aims to guide future research and improve clinical outcomes.

## 1 Introduction

### 1.1 Clinical burden and unmet need of threatened abortion

Threatened abortion (TA) is the most common complication in early pregnancy, affecting approximately 20-25% of all gestations (1, 2). Clinically defined by vaginal bleeding (with or without abdominal cramping) before 20 weeks of gestation in the presence of a viable intrauterine fetus, TA presents a significant diagnostic and therapeutic dilemma: while 75-85% of cases progress to uneventful pregnancy continuation, 15-25% culminate in irreversible miscarriage (3). This prognostic uncertainty not only induces considerable psychological distress in pregnant individuals but also contributes to suboptimal clinical management. This mismanagement ranges from the excessive use of empirical interventions, such as progesterone supplementation, to delayed recognition of progressive pregnancy loss (4).

Current clinical monitoring strategies primarily involve serial transvaginal ultrasound to evaluate fetal viability and placental location, alongside quantitative measurement of serum biomarkers, particularly β-hCG and progesterone (5). However, these approaches have inherent limitations: declining β-hCG levels or low progesterone concentrations reflect established trophoblast dysfunction rather than predicting the onset of pathological processes. Notably, serum β-hCG quantification fails to distinguish between transient trophoblast stress (reversible) and irreversible cellular damage, highlighting the critical need for mechanistic insights into TA pathogenesis to identify predictive biomarkers and targeted therapies.

### 1.2 Etiological hypotheses and the need for a unified model

The etiology of TA is widely recognized as multifactorial, with several key contributing factors extensively documented in the literature. These include endocrine disorders, such as luteal phase defect and thyroid dysfunction (6), impairments in maternal immune tolerance, exemplified by Th17/Treg cell imbalance and aberrant cytokine profiles (7, 8), thrombophilic conditions, notably antiphospholipid syndrome (9), and environmental stressors, including oxidative stress and nutrient deprivation (10). Although these studies have identified associations between specific phenotypic changes and TA, they predominantly remain descriptive and do not elucidate the dynamic sequence of molecular events that facilitate the transition from a reversible “at-risk” state to irreversible pregnancy loss.

A significant knowledge gap persists in understanding the upstream triggers of inflammation and the downstream effectors that sustain a maladaptive immune response. For instance, elevated levels of tumor necrosis factor-alpha (TNF-α) and skewed Th17/Treg ratios are consistently observed in TA (11, 12), yet the mechanisms by which these immune perturbations are initiated and maintained remain unclear. This lack of mechanistic understanding has hindered the development of targeted therapies, with current interventions limited to non-specific symptom management rather than addressing the root cause of TA.

### 1.3 The “danger signal” hypothesis: a novel framework for TA pathogenesis

**Figure 1:**
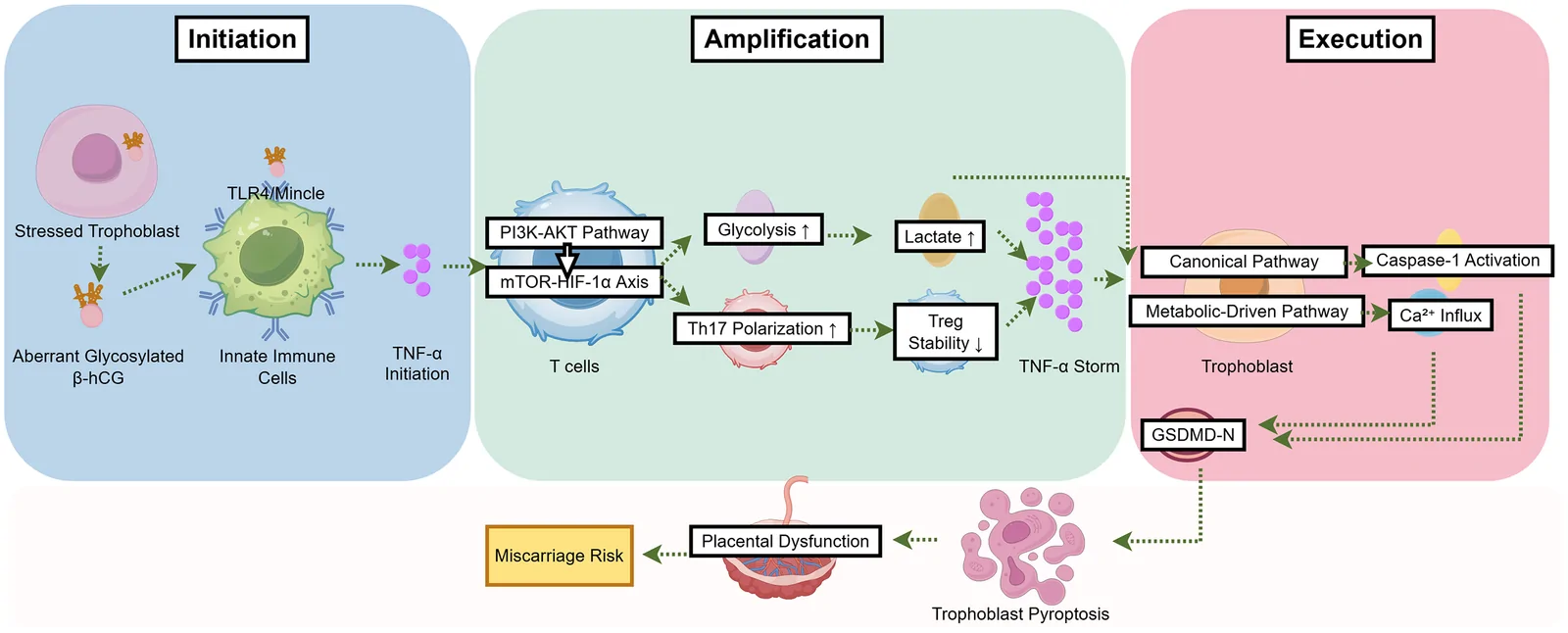
Schematic illustration of the three sequential stages in the pathogenic hypothesis of TA. The hypothesis delineates a stepwise cascade encompassing initiation, amplification, and execution, driven by core molecular and cellular events that ultimately lead to placental dysfunction and increased miscarriage risk. Drawn by FigDraw.

To address the existing gap in understanding TA pathogenesis, we propose the “Danger Signal” Hypothesis-a comprehensive and dynamic model that integrates glycobiology, immunometabolism, and cell death pathways to elucidate the progression of TA. This hypothesis suggests that TA is initiated by qualitative alterations in trophoblast-derived signals, as opposed to quantitative changes, which are misinterpreted by the maternal immune system as “danger” cues. This misinterpretation triggers a self-perpetuating cycle of inflammation and tissue damage. The hypothesis delineates three sequential stages (**Figure 1**): initiation through aberrant β-hCG glycosylation, amplification via TNF-α-mediated metabolic reprogramming, and execution by trophoblast pyroptosis. This review consolidates recent evidence to substantiate each stage of the hypothesis, examines its translational implications for the development of biomarkers and therapeutic interventions, and outlines future research directions to enhance TA management.

## 2 The initiation stage: aberrantly glycosylated β-hCG as a decoy danger signal

### 2.1 β-hCG glycosylation: a key regulator of its tolerogenic function

Human chorionic gonadotropin (hCG) is a heterodimeric glycoprotein consisting of an α-subunit, which is shared with luteinizing hormone, follicle-stimulating hormone, and thyroid-stimulating hormone, and a distinct β-subunit (β-hCG) (13). The β-subunit is extensively glycosylated, possessing four N-linked glycosylation sites (Asn13, Asn30, Asn63, Asn74) and one O-linked site (Ser121) (14). The composition and structural features of these glycan chains, including branching, sialylation, and fucosylation, are not merely structural modifications but are essential determinants of the biological activity and stability of β-hCG (15).

**Figure 2:**
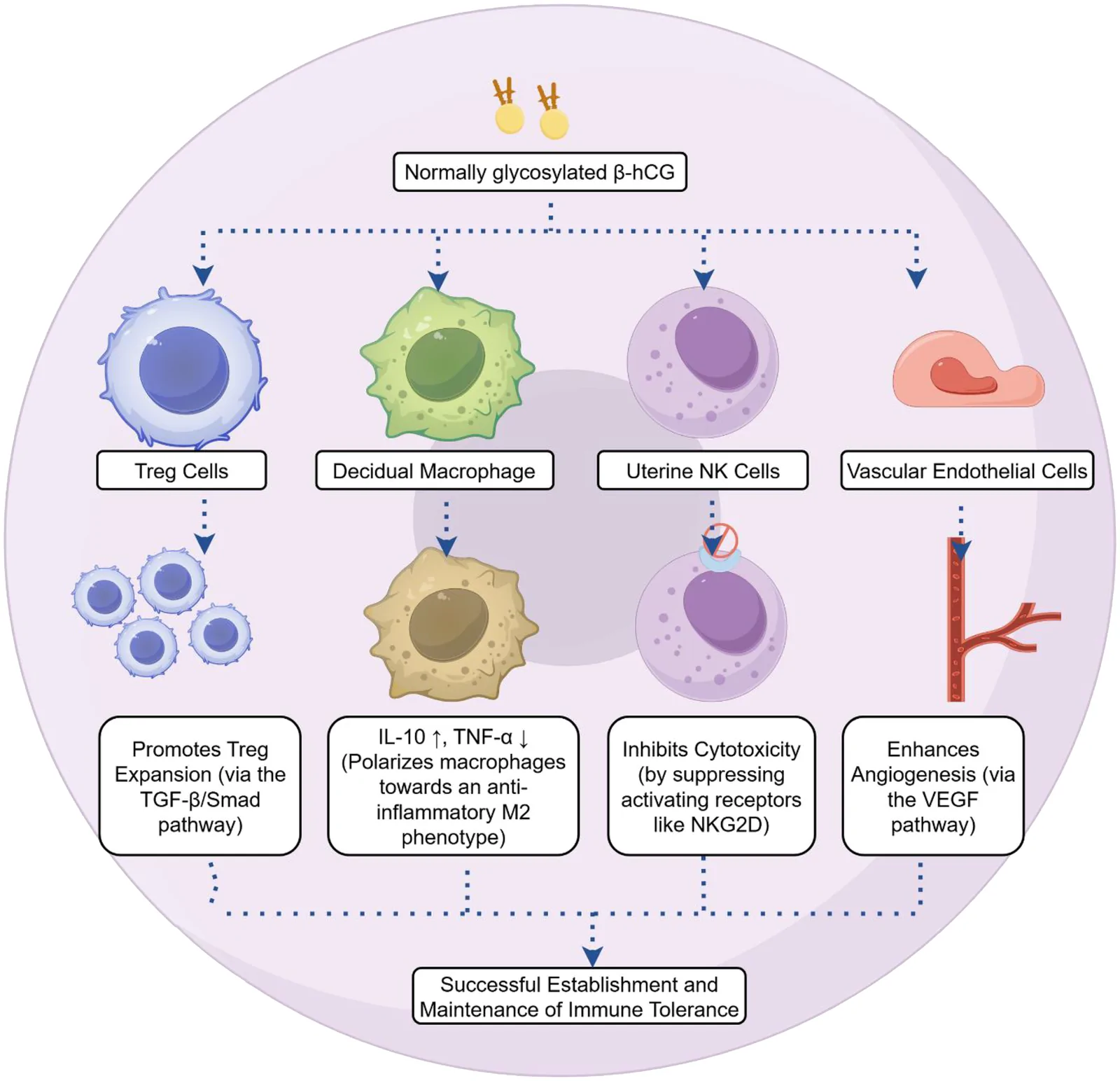
Mechanistic diagram illustrating the role of β-hCG with specific glycosylation features secreted by normal trophoblasts in establishing immune tolerance and promoting angiogenesis during pregnancy. Drawn by FigDraw.

During normal early pregnancy, trophoblasts secrete β-hCG with a specific glycosylation profile that facilitates maternal immune tolerance towards the semi-allogeneic fetus. Proper glycosylation of β-hCG induces tolerogenic effects through several mechanisms (**Figure 2**): (1) it promotes the expansion of regulatory T cells (Tregs) via activation of the TGF-β/Smad signaling pathway (16); (2) it polarizes decidual macrophages towards the anti-inflammatory M2 phenotype by upregulating IL-10 and downregulating TNF-α (17); (3) it suppresses the cytotoxicity of natural killer (NK) cells by inhibiting the expression of activating receptors, such as NKG2D (17, 18); (4) enhancing angiogenesis at the maternal-fetal interface via activation of the VEGF pathway (19). Collectively, these functions establish β-hCG as a central mediator of pregnancy maintenance, with its glycosylation profile serving as a “molecular signature” of trophoblast health.

### 2.2 Stress-induced alterations in β-hCG glycosylation and DAMP transformation

Trophoblasts exhibit a high susceptibility to cellular stressors, which are prevalent during early pregnancy and become exacerbated under pathological conditions. Principal stressors include oxidative stress, arising from inadequate placental perfusion; endoplasmic reticulum (ER) stress, resulting from impaired protein folding; and nutrient deprivation, such as deficiencies in glucose or amino acids (20, 21). These stressors disrupt the enzymatic machinery involved in glycosylation, including glycosyltransferases (e.g., GnT-IV, GnT-V) and glycosidases, leading to the production of β-hCG with immature or aberrant glycan structures (22, 23).

A well-characterized aberrant glycoform is hyperglycosylated hCG (hCG-h). During normal pregnancy, hCG-h is the predominant form secreted by invasive cytotrophoblasts in the first 6–8 weeks of gestation, where it plays a critical role in placental implantation (24). Prospective studies have consistently shown that higher hCG-h/total hCG ratios at implantation are associated with ongoing pregnancies (25). Beyond this window, the syncytiotrophoblast becomes the dominant hCG source, and glycosylation normally shifts toward less branched, fully sialylated isoforms. Persistent hCG-h production by syncytiotrophoblasts thereafter represents a maladaptive reversion to a primitive glycosylation pattern, indicative of trophoblast stress, and has been linked to early miscarriage (26, 27).

The structural anomalies of hCG-h are defined by two key alterations (28). First, there is a significant extension and complexification of O-linked glycans on the β-subunit’s C-terminal peptide (CTP), where larger 6-sugar structures become predominant, contrasting sharply with the simpler cores of native hCG. Second, the N-linked glycans exhibit a marked increase in tri-antennary (branched) structures, a change functionally analogous to the upregulation of GnT-V. Concurrently, a significant loss of terminal sialic acid residues exposes underlying sugar motifs, such as galactose and N-acetylglucosamine (GlcNAc), which are typically “capped” and hidden. These changes are functionally consistent with upregulated GnT-V activity and impaired sialyltransferase function under stress conditions (23).

These structural shifts fundamentally alter receptor engagement. The highly branched N-glycans structurally resemble known TLR4 ligands such as lipopolysaccharide (29), potentially promoting aberrant TLR4/NF-κB activation (30). Concurrently, exposed terminal sugars (Gal, GlcNAc) resemble ligands for C-type lectin receptors such as Mincle (31), while enhanced core fucosylation via FUT8 may also engage lectin receptors (32). Although direct biophysical evidence of β-hCG binding to TLR4/Mincle remains limited, the striking structural homology to pathogen-associated molecular patterns (PAMPs) strongly implies promiscuous engagement with these PRRs (33). Consequently, the glycan profile shifts from tolerogenic lectins (Galectin-1, DC-SIGN) toward inflammatory PRRs (TLR4, Mincle), initiating an inflammatory cascade.

While existing *in vitro* models, such as hypoxia-reoxygenation in HTR-8/SVneo cells, have demonstrated that oxidative stress can enhance glycosyltransferase activity, including GnT-V, thereby suggesting an influence on β-hCG glycosylation, there is still an absence of direct evidence connecting oxidative stress, such as H_2_O_2_ treatment, in BeWo cells or primary trophoblasts to increased hCG-h secretion or reduced sialyltransferase activity (34, 35). Establishing this causal link between stress and specific glycosylation phenotypes remains a key step in confirming the upstream trigger of the “Danger Signal” hypothesis.

**Figure 3:**
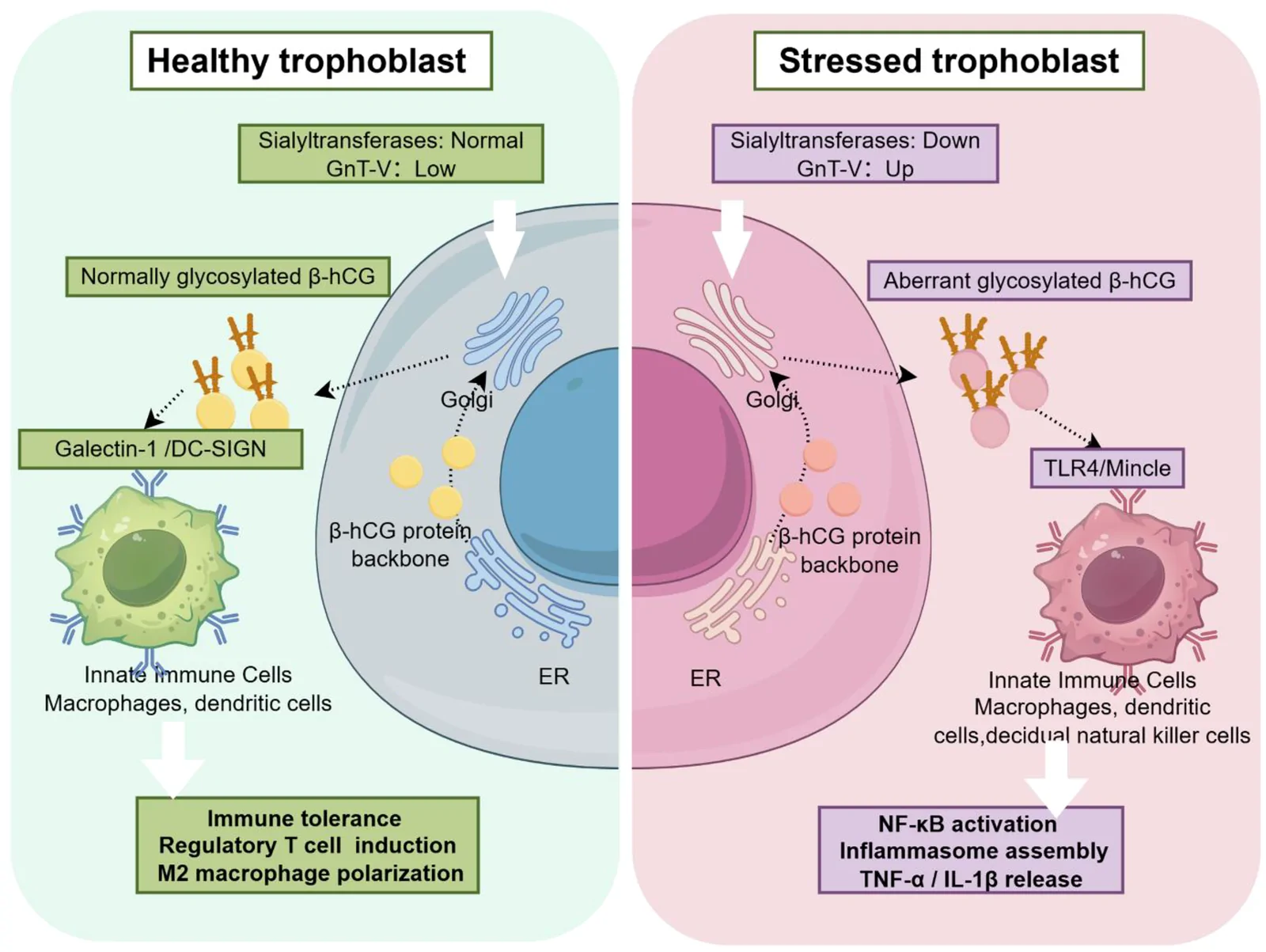
Glycosylation-mediated immune modulation by β-hCG at the fetomaternal interface. Healthy trophoblasts secrete normally glycosylated β-hCG that binds tolerogenic lectins (Galectin-1/DC-SIGN) to maintain immune tolerance. Stressed trophoblasts produce aberrantly glycosylated β-hCG, which fails to engage tolerogenic receptors but instead activates pro-inflammatory PRRs (TLR4/Mincle). This aberrant glycoform drives NF-κB signaling and inflammasome assembly in innate immune cells (macrophages, DCs, dNK cells), initiating a pathological inflammatory cascade. ER: Endoplasmic Reticulum; Golgi: Golgi apparatus. Drawn by FigDraw.

As summarized in **Figure 3**, we hypothesize that stress-induced aberrant glycosylation converts β-hCG from a tolerogenic signal into a DAMP. Aberrant β-hCG glycoforms engage TLR4 on decidual macrophages, activating NF-κB, and Mincle on DCs, triggering inflammasome activation. This inappropriate PRR engagement causes the maternal immune system to erroneously recognize aberrant β-hCG as a danger signal, initiating a pathological cascade.

## 3 The amplification stage: TNF-α storm and T cell metabolic reprogramming

### 3.1 TNF-α: the central amplifier of inflammation

The recognition of aberrant β-hCG by innate immune cells induces the secretion of pro-inflammatory cytokines, with TNF-α playing a pivotal role in the amplification phase. In addition to decidual macrophages and dendritic cells (DCs), the abundant decidual natural killer (dNK) cells at the implantation site may directly sense the aberrant glycoforms of β-hCG via surface PRRs (36). We hypothesize that this interaction could potentially trigger dNK cells degranulation and the secretion of TNF-α and IFN-γ, contributing to the initial propagation of the inflammatory cascade. Consequently, TNF-α levels in both serum and decidual tissue are markedly elevated in women with threatened abortion (TA) who subsequently experience miscarriage (37, 38). A meta-analysis comprising 11 studies (n=1371) revealed that the standardized mean difference in TNF-α levels was 2.82 units (95% confidence interval: 1.57-4.06) in patients who experienced miscarriage compared to those with ongoing pregnancies (*P* < 0.0001) (39).

**Figure 4:**
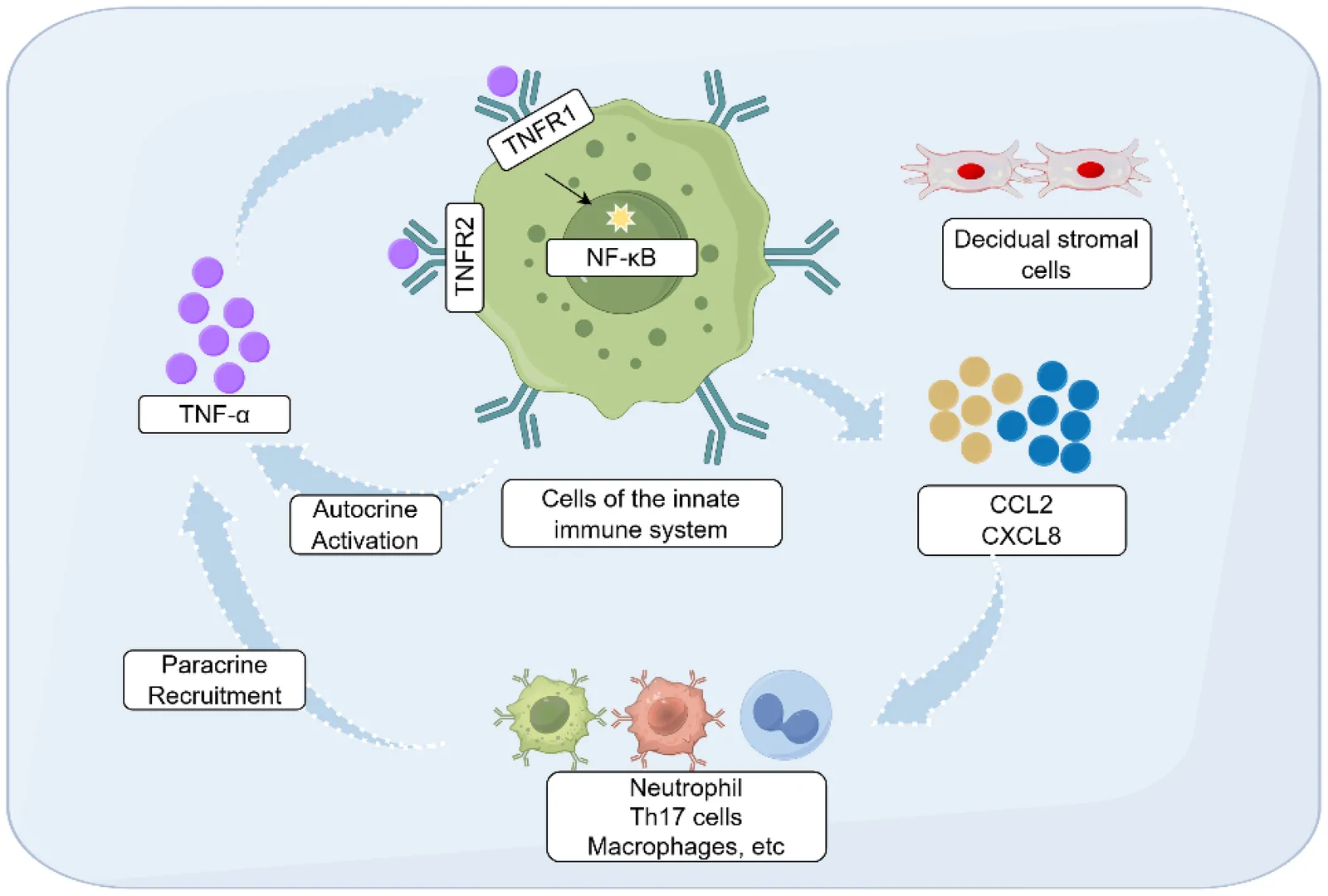
Schematic illustration of the two key mechanisms by which TNF-α drives a self -perpetuating inflammatory loop. TNF-α can bind to TNFR1 and TNFR2 on the surface of immune cells to activate NF-κB signaling pathway, achieving autocrine activation of innate immune cells. Meanwhile, activated innate immune cells secrete chemokines such as CCL2 and CXCL8 to recruit immune cells including neutrophils, Th17 cells, and macrophages from the periphery (paracrine recruitment), participating in the initiation and regulation of immune responses. Drawn by FigDraw.

TNF-α perpetuates an inflammatory feedback loop through two principal mechanisms, as illustrated in **Figure 4**: (1) Autocrine activation, wherein TNF-α binds to its receptors (TNFR1/2) on innate immune cells, subsequently activating the NF-κB pathway, which enhances the transcription of TNF-α (40, 41); (2) Paracrine recruitment, whereby TNF-α stimulates the expression of chemokines, such as CXCL8 and CCL2, which attract additional pro-inflammatory cells, including neutrophils and Th17 cells, to the maternal-fetal interface (42, 43). This process culminates in a “TNF-α storm, “ characterized by a nonlinear escalation in local TNF-α concentrations that surpasses the capacity of endogenous anti-inflammatory mechanisms, such as soluble TNFRs and IL-10, to mitigate (44, 45).

Importantly, the shift from physiological TNF-α, which is integral to normal placental remodeling, to a pathological TNF-α storm is delineated by a critical threshold. Although a universally accepted absolute concentration threshold for TNF-α in early pregnancy remains unestablished—largely due to inter-individual variability, methodological differences across assays, and the transient nature of cytokine kinetics, a functional threshold​ can be conceptually defined. Rather than relying on absolute serum levels, this threshold is better characterized by the balance between pro-inflammatory and regulatory mediators. Specifically, a sustained shift in the TNF-α/IL-10 ratio or an elevated free TNF-α to soluble receptor (sTNFR2) ratio reflects a transition from physiological immune adaptation to a pathological inflammatory state (12, 39). This functional tipping point corresponds to the “point of no return, “​ wherein transient NF-κB signaling gives way to sustained activation, driving trophoblast apoptosis and establishing a self-perpetuating inflammatory loop (46). Definitive quantitative cutoffs for clinical application await validation in large, standardized prospective cohorts.

### 3.2 Immunometabolic reprogramming: the mechanism of irreversible inflammation

The “Danger Signal” Hypothesis presents a novel perspective by identifying T cell metabolic reprogramming as the mechanism through which TNF-α maintains the immune system in a pro-inflammatory state. The functionality of immune cells is intricately linked to their metabolic pathways: pro-inflammatory T cells, such as Th1 and Th17, predominantly utilize aerobic glycolysis (known as the Warburg effect) to facilitate rapid energy production and biomass synthesis. In contrast, Tregs rely on oxidative phosphorylation (OXPHOS) and fatty acid oxidation (FAO) to exert their suppressive functions (47, 48).

**Figure 5:**
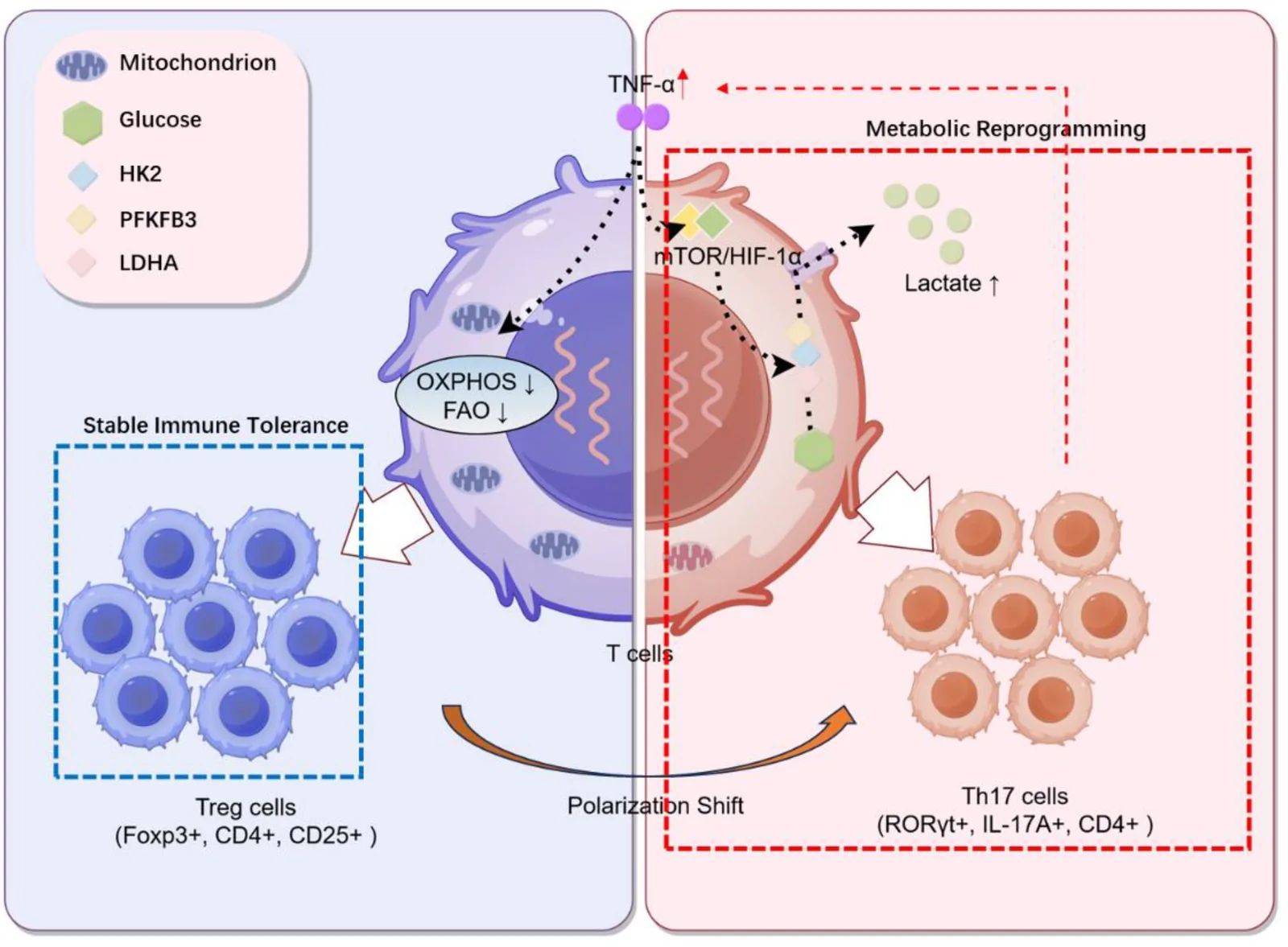
TNF-α-mediated metabolic reprogramming in T cells. Left-sided Treg cells (Treg cells, Foxp3^+^, CD4^+^, CD25^+^) rely on mitochondrial OXPHOS and FAO to maintain stable immune tolerance. Under the influence of factors such as TNF-α, T cells undergo a polarization shift, driving metabolic reprogramming through the mTOR/HIF-1α pathway, enhancing the activity of HK2, PFKFB3, and LDHA, shifting glucose metabolism toward glycolysis, and increasing lactate levels, ultimately differentiating into right-sided Th17 cells (RORγt^+^, IL-17A^+^, CD4^+^). Drawn by FigDraw.

TNF-α serves as a pivotal regulator of this metabolic equilibrium by promoting a shift in T cell metabolism towards glycolysis via activation of the mTOR-HIF-1α axis (**Figure 5**). Initially, the binding of TNF-α to TNFR1 triggers the PI3K-AKT signaling pathway, leading to the phosphorylation and activation of mTOR, a critical metabolic sensor that enhances glycolysis by upregulating the expression of glucose transporters (e.g., GLUT1) and glycolytic enzymes (e.g., hexokinase 2, lactate dehydrogenase A) (49, 50). Secondly, the activation of the mechanistic target of rapamycin (mTOR), in conjunction with TNF-α-induced production of reactive oxygen species (ROS), leads to the stabilization of hypoxia-inducible factor-1 alpha (HIF-1α) (51). HIF-1α is a transcription factor that promotes the expression of glycolytic genes, such as 6-phosphofructo-2-kinase/fructose-2, 6-bisphosphatase 3 (PFKFB3), and transcription factors specific to Th17 cells, such as retinoic acid-related orphan receptor gamma t (RORγt) (52). Simultaneously, TNF-α compromises mitochondrial function by inducing ROS-mediated damage to mitochondrial DNA (mtDNA) and inhibiting the activity of complex I within the electron transport chain (53, 54), This results in a reduction of oxidative phosphorylation (OXPHOS) and fatty acid oxidation (FAO), thereby undermining the stability and function of regulatory T cells (Tregs). The “point of no return” in the progression of TA is characterized by the completion of this metabolic reprogramming; once T cells transition from OXPHOS to glycolysis, the pro-inflammatory phenotype becomes self-perpetuating through metabolic-transcriptional and metabolic-cytokine feedback mechanisms.

## 4 Trophoblast pyroptosis: the execution stage of inflammatory demise

### 4.1 Mechanisms of trophoblast pyroptosis in TA

While apoptosis (programmed, non-inflammatory cell death) has long been recognized in trophoblast dysfunction (55), recent evidence identifies pyroptosis as the dominant cell death mechanism driving irreversible tissue damage in TA. Pyroptosis is mediated by gasdermin (GSDM) family proteins, particularly gasdermin D (GSDMD), which form pores in the plasma membrane upon cleavage by inflammatory caspases (caspase-1/4/5/11) (56). The formation of pores in the cellular membrane results in cellular swelling, the release of intracellular components—including damage-associated molecular patterns (DAMPs) and pro-inflammatory cytokines—and the subsequent amplification of the inflammatory response, which is markedly different from the silent clearance of apoptotic cells mediated by phagocytosis (57).

**Figure 6:**
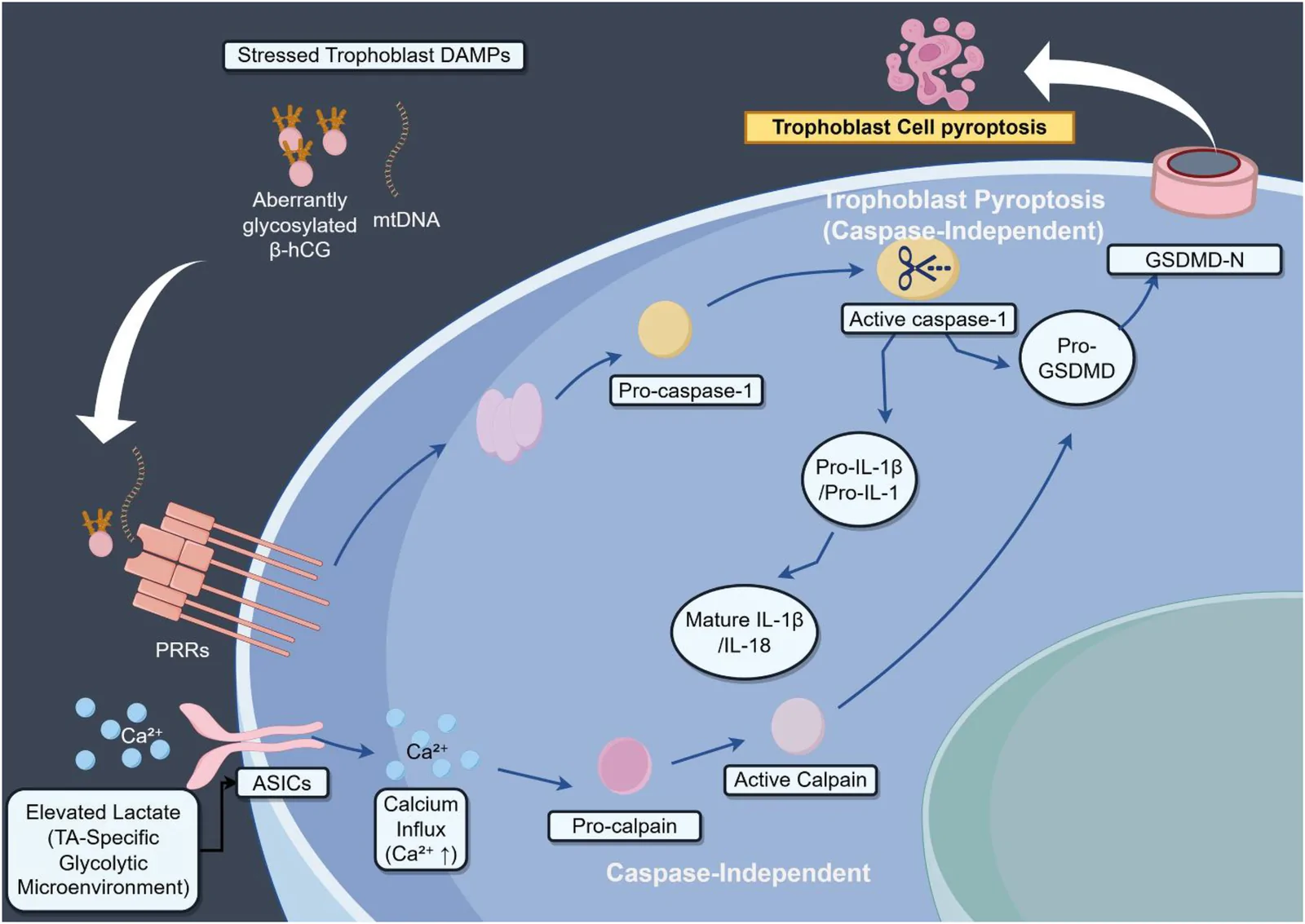
Two converging pathways of trophoblast pyroptosis in TA. In the trophoblast-specific glycolytic microenvironment, elevated lactate activates ASICs channels, triggering calcium influx (Ca^2+^↑). Meanwhile, DAMPs, such as aberrantly glycosylated β-hCG and mtDNA released by stressed trophoblast cells activate PRRs. The calcium influx activates calpain, and PRRs activation induces the maturation of pro-caspase-1 into active caspase-1. Active caspase-1 not only promotes the maturation of pro-IL-1β/IL-18 into active IL-1β/IL-18 but also activates pro-GSDMD to form GSDMD-N, ultimately leading to caspase-independent pyroptosis of trophoblast cells and the release of cellular contents. Drawn by FigDraw.

In the context of TA, trophoblast pyroptosis is triggered by two converging pathways: the canonical inflammasome pathway and the metabolically driven pathway (**Figure 6**). The canonical pathway is activated by DAMPs released from stressed trophoblasts, such as aberrant β-hCG and mtDNA, as well as by pathogens, including those responsible for intrauterine infections. These stimuli activate nucleotide-binding oligomerization domain (NOD)-like receptors (NLRs), including NLRP3, which assemble into inflammasomes—multiprotein complexes that facilitate the recruitment and activation of caspase-1 (58). Once activated, caspase-1 cleaves pro-IL-1β and pro-IL-18 into their mature forms and processes GSDMD into its active N-terminal fragment (GSDMD-N), thereby initiating pyroptosis (59).

The metabolically driven pathway, which is specific to TA, is triggered by the glycolytic microenvironment established during the amplification phase. Elevated lactate levels (a byproduct of T cell glycolysis) reduce intracellular pH, activating acid-sensing ion channels (ASICs) on trophoblasts (60). ASIC activation induces calcium influx, which activates calpain proteases—enzymes that directly cleave GSDMD into GSDMD-N, thus providing a non-canonical, caspase-independent route to pyroptosis. This calpain-mediated pathway operates in parallel with the canonical inflammasome pathway, explaining why pyroptosis persists even in the absence of canonical inflammasome activation (61). This caspase-independent pathway explains why pyroptosis persists even in the absence of canonical inflammasome activation.

### 4.2 Pyroptosis-mediated vicious cycle of inflammation

**Figure 7:**
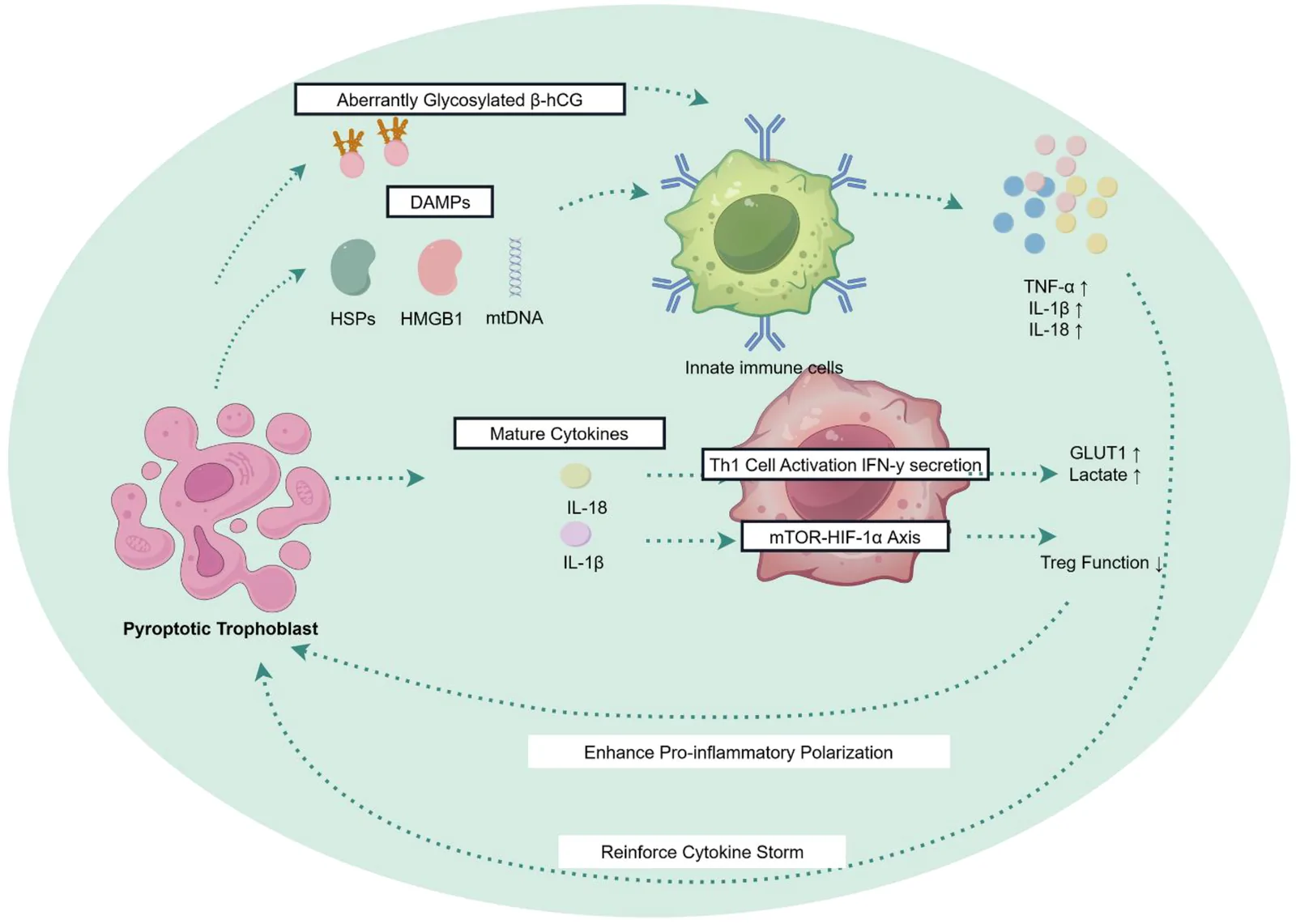
Schematic diagram illustrating the vicious cycle of inflammation and tissue damage during the execution stage of TA. Drawn by FigDraw.

The execution stage completes the pathogenic cascade by creating a vicious cycle of inflammation and tissue damage (**Figure 7**). Pyroptotic trophoblasts release three classes of pro-inflammatory mediatorsthat perpetuate the pathological response: DAMPs, mature cytokines, and aberrant glycan fragments.

First, DAMPs such as mtDNA, high-mobility group box 1 (HMGB1), and heat shock proteins (HSPs) are released into the decidual microenvironment upon trophoblast pyroptosis (62). These DAMPs interact with PRRs on innate immune cells; for instance, mtDNA activates Toll-like receptor 9 (TLR9), while HMGB1 engages Toll-like receptor 4 (TLR4), thereby further stimulating the secretion of TNF-α and IL-1β (63). A study by Huang et al. (64) demonstrated that HMGB1 released from pyroptotic trophoblasts elevates the production of IL-18, IL-1β, and TNF-α by decidual macrophages, thereby reinforcing the cytokine storm characteristic of the amplification stage.

Furthermore, mature pro-inflammatory cytokines (IL-1β and IL-18), cleaved during pyroptosis, enhance the metabolic skewing and pro-inflammatory polarization of T cells. IL-1β activates the mTOR- HIF-1αaxis in T cells, leading to increased expression of glucose transporter 1 (GLUT1) and lactate production (65). Concurrently, IL-18 promotes interferon-gamma (IFN-γ) secretion by Th1 cells, which suppresses Treg function and further disrupts immune tolerance. Clinical evidence corroborates these findings: a study involving 44 patients with TA revealed that serum IL-1β levels were significantly higher in those who experienced miscarriage compared to those with ongoing pregnancies (*P* < 0.001) (66).

Third, aberrantly glycosylated β-hCG released from pyroptotic trophoblasts is present in elevated concentrations, perpetuating the activation of TLR4 and Mincle on innate immune cells (30). This process sustains the initial DAMP signal, thereby ensuring the persistence of the inflammatory response even as trophoblasts undergo cell death. Collectively, this cycle results in extensive placental dysfunction: the pyroptosis of syncytiotrophoblasts compromises the transport of nutrients and oxygen to the fetus, while the pyroptosis of cytotrophoblasts disrupts placental invasion and angiogenesis (67).

## 5 Translational applications: biomarkers, therapeutics, and challenges

### 5.1 Stage-specific biomarkers for TA prognosis

The “Danger Signal” Hypothesis facilitates the identification of stage-specific biomarkers that address the limitations inherent in current clinical assessments, such as serum β-hCG and ultrasound, which primarily reflect established dysfunction rather than predictive pathological processes. These biomarkers not only enhance prognostic accuracy but also inform targeted interventions by delineating the pathological stage of TA.

For the initiation stage, the ratio of hCG-h to total hCG serves as a robust predictive marker. A prospective study conducted by Salas et al. demonstrated that the median levels of hCG-h and total hCG in pregnancies with normal outcomes were significantly higher than those in pregnancies resulting in abortion among TA patients (68). Additional glycan-specific markers, such as reduced β-hCG sialylation (quantified by mass spectrometry) and increased α1, 6-fucosylation (identified through Lens culinaris agglutinin binding), have been associated with trophoblast stress and maternal-fetal immune tolerance (69, 70).

During the amplification stage, biomarkers indicative of metabolic reprogramming exhibit high prognostic value. In a study conducted by Wu et al. involving 60 participants, it was found that Galectin-1 expression levels were significantly lower in patients experiencing spontaneous abortion compared to those undergoing induced abortion during normal early pregnancy (71). Furthermore, flow cytometric analysis of circulating T cells revealed a reduced percentage of CD4+CD25+Foxp3+ regulatory T cells in the spontaneous abortion group compared to the induced abortion group during normal early pregnancy (0.77 ± 0.31 vs. 1.00 ± 0.35, *P* < 0.05). These results suggest that the downregulation of Galectin-1 and modifications in regulatory T cells may play a pivotal role in the pathogenesis of spontaneous abortion (72). Additionally, lactate metabolism is critical in decidual macrophage-mediated miscarriage, and inhibiting lactate uptake has been shown to ameliorate abortion-prone models (73). Based on the aforementioned studies, the combination of these two markers with β-hCG may serve as biomarkers for TA.

For the execution stage, circulating GSDMD-N fragments (detected via ELISA or Western blot) and serum IL-1β levels mark irreversible pyroptosis. In the villous tissues of patients with recurrent spontaneous abortion (RSA), the protein levels of GSDMD-N fragments and IL-1β are significantly higher than those in the healthy control group (58). A retrospective analysis of 108 patients with threatened abortion has demonstrated that IL-1β is a risk factor for threatened abortion (74). Similarly, a nested case-control study conducted by Hu et al. revealed that the serum IL-1β level in the live birth group was significantly higher than that in the abortion group (*P* < 0.001), and serum IL-1β level was significantly negatively correlated with the risk of abortion (75).

### 5.2 Targeted therapeutic strategies

The mechanistic understanding of the β-hCG-TNF-α-T cell axis facilitates the development of targeted therapies that address specific stages of TA progression, thereby advancing beyond non-specific interventions such as progesterone supplementation.

In the initial phase, the primary strategy involves restoring normal β-hCG glycosylation by reducing trophoblast stress. Antioxidants, such as N-acetylcysteine (NAC), play a crucial role in mitigating oxidative stress, which is a significant contributor to abnormal glycosylation. A clinical study with 166 women experiencing recurrent miscarriage and elevated oxidative stress markers demonstrated that NAC supplementation (600 mg/day) resulted in a 72% success rate in sustaining pregnancy, compared to a placebo group (76). The effectiveness of NAC intervention was further corroborated in a threatened abortion model (77). Additionally, endoplasmic reticulum stress chaperones, such as 4-phenylbutyrate (4-PBA), enhance glycosylation by restoring glycosyltransferase activity. Research conducted by Liong et al. revealed that 4-phenylbutyric acid alleviates endoplasmic reticulum stress and inflammatory responses, thereby safeguarding gestational tissues and offering direct clinical mechanistic insights for threatened abortion (78).

In the amplification phase, the inhibition of TNF-α and metabolic reprogramming are of paramount importance. Anti-TNF-α agents, including etanercept (a soluble TNFR2 fusion protein), have been shown to mitigate cytokine storms. A randomized controlled trial (n=188) demonstrated that the group receiving etanercept (25 mg/week for 4 weeks) exhibited a significant reduction in TNF-α levels and achieved a healthy live birth rate of 89.47%, significantly surpassing the 72.04% observed in the placebo group (*P* = 0.01) (79). In terms of metabolic modulation, metformin, an AMPK activator, counteracts T cell glycolysis by inhibiting mTOR. Treg cells are characterized by a metabolic profile with elevated GLUT1 expression, and metformin can influence cellular metabolism by targeting this characteristic (80).

During the execution stage, inhibiting pyroptosis is crucial to prevent irreversible trophoblast loss. GSDMD inhibitors, such as necrosulfonamide, bind to GSDMD-N to inhibit pore formation. *In vitro* experiments have confirmed that necrosulfonamide (10 μmol/L) effectively targets the death pathway in trophoblast-related cells (81). For caspase-dependent pyroptosis, NLRP3 inhibitors like MCC950 impede inflammasome activation, thereby reducing IL-1β secretion and LDH release, and enhancing pregnancy-related outcomes in stressed trophoblast cells (82).

### 5.3 Unmet challenges in translation

Despite encouraging preclinical and early clinical findings, several challenges must be addressed to facilitate the widespread clinical adoption of these therapies.

Firstly, the safety of these interventions during pregnancy remains a significant concern. Many candidate therapies, such as anti-TNF-α agents and GSDMD inhibitors, lack comprehensive long-term safety data in humans. Beyond inflammatory and cell death inhibitors, metabolic interventions represented by metformin also carry unique safety risks when applied to early threatened abortion. Metformin is widely used for gestational diabetes and polycystic ovary syndrome in pregnancy, but its off-label use for immune metabolic reprogramming in threatened abortion lacks dedicated safety verification. Early pregnancy is a critical window for embryonic organogenesis; metformin-mediated systemic glycolysis inhibition may interfere with physiological energy metabolism of trophoblasts and embryonic cells. A recent primate study further confirmed these concerns. When human-equivalent doses of metformin were given to rhesus monkeys within 30 days of conception, the drug exhibited prominent bioaccumulation in multiple fetal tissues and gestational compartments. The exposed fetuses presented diminished skeletal muscle and retroperitoneal fat, significantly decreased birth weight, and abnormal renal development including disorganized glomerular structure and increased cortical thickness. Notably, these adverse outcomes were not attenuated by maternal dietary patterns (83). Moreover, existing clinical studies mainly focus on patients with pre-gestational metabolic disorders, and there is a paucity of large cohort data clarifying its risk of teratogenicity, adverse fetal weight and long-term metabolic sequelae in fetuses exposed during the first trimester (84).For instance, Etanercept is classified as FDA Category B; however, data regarding fetal exposure and long-term outcomes in infants are limited (85). Future randomized controlled trials (RCTs) should incorporate extended follow-up periods (≥2 years) for infants exposed to these interventions to evaluate neurodevelopmental and metabolic outcomes comprehensively.

Secondly, there is a need to improve the accessibility of biomarkers.

Currently, most biomarkers, such as decidual lactate and placental GSDMD-N, require invasive sampling methods (e.g., decidual biopsy) or specialized techniques (e.g., mass spectrometry), which restrict their clinical applicability (86, 87). To overcome limited biomarker accessibility, we herein present a tiered translational framework to translate non-invasive detection assays into clinical use. First, laboratory testing platforms will be converted to point-of-care formats, including lectin-based lateral flow immunoassays (LFAs) for rapid bedside measurement of urinary β-hCG glycoforms such as hCG-h (68) and standardized nanoparticle tracking analysis (NTA) to quantify plasma extracellular vesicle (EV)-derived GSDMD-N and obviate tissue biopsy (88). Second, analytical validation against gold-standard approaches including glycan mass spectrometry and GSDMD-N Western blot will be performed in a 200-subject pilot cohort to establish diagnostic sensitivity, specificity and cut-off thresholds. Last, multicenter prospective evaluation in >1000 threatened abortion patients with serial baseline, 4-week and 8-week biospecimens will define the biomarkers’ predictive performance for pregnancy outcomes, ensuring robust clinical applicability and broad accessibility.

Threatened abortion presents prominent heterogeneity, with patient subgroups modulated by divergent stressors including oxidative stress and intrauterine infection; anti-TNF-α agents exert superior therapeutic effects on infection-related cases, while antioxidants are more suitable for phenotypes induced by oxidative stress (89, 90). Though the proposed β-hCG-TNF-α-T cell axis unifies the pathogenic cascade of threatened abortion, multiple upstream triggers covering oxidative stress, endoplasmic reticulum stress and intrauterine infection converge on placental tissues to elicit the danger signal phenotype with unique kinetic features and biomarker signatures. Infectious stimuli activate pathway amplification through TLR4/Mincle recognition of pathogen-associated molecular patterns and trigger an early, sharp rise in TNF-α, whereas oxidative stress subtly disturbs glycosyltransferase activity at the pathogenic initiation phase and gradually accumulates abnormal β-hCG glycoforms, highlighting the requirement for stratified biomarker detection. We therefore establish a Dual-Layer Biomarker Strategy: the first layer conducts etiology-targeted screening via Gardnerella vaginalis quantification and 8-OHdG measurement to distinguish primary pathogenic insults, and the second layer monitors distinct stages of the core axis using the hCG-h ratio to reflect initiation and TNF-α/IL-1β to indicate amplification. This two-tiered strategy enables stage- and etiology-matched treatment and avoids improper anti-inflammatory administration when infection clearance is prioritized, and composite panels integrating the hCG-h ratio, IL-1β concentrations and microbiome profiling should be adopted in subsequent clinical trials to stratify patients and improve therapeutic responsiveness (91).

## 6 Future perspectives and conclusion

### 6.1 Priority research directions

To enhance the understanding and management of TA, future research should prioritize three key areas.

Firstly, single-cell and spatial omics approaches will elucidate cell-specific mechanisms (92). Techniques such as single-cell RNA sequencing (scRNA-seq) of decidual tissues will facilitate the identification of T cell subsets, such as Th17 and Treg, that are most susceptible to metabolic reprogramming, as well as trophoblast populations, including syncytiotrophoblasts and extravillous trophoblasts, that are prone to pyroptosis. Spatial transcriptomics is poised to enhance our understanding of the distribution of inflammatory mediators, such as TNF-α and lactate, as well as PRRs like TLR4 and Mincle, within the maternal-fetal interface. This approach is expected to illuminate regional variations in pathological processes, potentially elucidating why certain cases of TA advance to miscarriage while others resolve.

Second, microbiome-immune interactions represent an understudied area (93). The composition of the vaginal and uterine microbiomes plays a critical role in modulating maternal immune tolerance, with dysbiosis-characterized by an increase in Gardnerella vaginalis and a decrease in Lactobacillus crispatus-being linked to TA. Future research should focus on elucidating the regulatory effects of microbiome-derived metabolites, such as short-chain fatty acids, on β-hCG glycosylation and trophoblast pyroptosis. Such studies could pave the way for microbiome-based therapeutic interventions, including the use of Lactobacillus probiotics, to restore immune tolerance.

Third, longitudinal multi-center studies will validate the “Danger Signal” Hypothesis. These studies should involve prospective cohorts with repeated sampling of serum, urine, and vaginal swabs at intervals of 4–6 weeks to monitor the temporal progression of changes in β-hCG glycosylation, metabolic reprogramming, and pyroptosis in TA (94). These studies will refine biomarker thresholds for each stage and enable the development of a “TA progression score” to guide clinical decision-making.

### 6.2 Conclusion

This review introduces a comprehensive “Danger Signal” Hypothesis, which delineates the pathogenesis of TA into three sequential and interconnected stages: initiation through aberrant β-hCG glycosylation, amplification via TNF-α-mediated T cell metabolic reprogramming, and execution by trophoblast pyroptosis. By synthesizing insights from glycobiology, immunometabolism, and cell death research, this model transcends mere descriptive associations, offering an explanation for the dynamic transition from reversible trophoblast stress to irreversible pregnancy loss—a significant gap in current TA research.

The translational implications of this framework are significant. Stage-specific biomarkers, such as the hCG-h ratio, GLUT1^+^ T cells, and GSDMD-N, enhance prognostic accuracy, while targeted therapies, including NAC, etanercept, and NSA, address the underlying causes of TA rather than merely alleviating symptoms. Despite ongoing challenges in safety validation, biomarker accessibility, and patient stratification, the proposed hypothesis offers a strategic pathway for the application of precision medicine in the management of TA.

Ultimately, advancing our understanding of the β-hCG-TNF-α-T cell axis is anticipated not only to improve clinical outcomes for TA but also to provide insights into other pregnancy complications, such as preeclampsia, which share similar mechanisms involving inflammation and trophoblast dysfunction. This cross-disease relevance highlights the broader impact of TA research on maternal and fetal health and underscores the necessity for continued investment in mechanistic and translational studies.
